# Transferability of Molecular Potentials for 2D Molybdenum Disulphide

**DOI:** 10.3390/ma14030519

**Published:** 2021-01-21

**Authors:** Marcin Maździarz

**Affiliations:** Institute of Fundamental Technological Research Polish Academy of Sciences, 02-106 Warsaw, Poland; mmazdz@ippt.pan.pl; Tel.: +48-22-826-12-81

**Keywords:** 2D materials, MoS2, molecular potentials, DFT, elastic constants, phonons

## Abstract

An ability of different molecular potentials to reproduce the properties of 2D molybdenum disulphide polymorphs is examined. Structural and mechanical properties, as well as phonon dispersion of the 1H, 1T and 1T’ single-layer MoS${}_{2}$ (SL MoS${}_{2}$) phases, were obtained using density functional theory (DFT) and molecular statics calculations (MS) with Stillinger-Weber, REBO, SNAP and ReaxFF interatomic potentials. Quantitative systematic comparison and discussion of the results obtained are reported.

## 1. Introduction

Group 6 transition metal dichalcogenide (G6-TMD) two-dimensional (2D) nanomaterials [1], and especially single-layer molybdenum disulphide (SL MoS${}_{2}$), are probably the second most studied 2D materials following graphene [2]. The major disadvantages of graphene are the lack of a band gap in the electronic spectrum, its susceptibility to oxidative environments and that it has some toxic properties. That is why scientists and engineers, beyond ordinary human curiosity, have begun to look for materials free of these deficiencies [3,4].

Both synthetic and natural bulk transition metal dichalcogenides have layered structures with two primary distinguished allotropic forms, 2H and 3R, belonging to the hexagonal crystal family, but differing in a sequence of arrangement. Strong triple layers of metal-sulphur-metal are weakly bounded by the van der Waals forces, similar to graphene in graphite [1].

Three polymorphs of single-layer molybdenum disulphide have been synthesised, namely the most thermodynamically stable semiconducting 1H-MoS${}_{2}$, semimetallic 1T’-MoS${}_{2}$ and metastable metallic 1T-MoS${}_{2}$ [5]. In 1H-MoS${}_{2}$ structural phase, the S and Mo atoms are stacked in an A-B-A order, the 1T-MoS${}_{2}$ dynamically unstable phase has an A-B-C stacking, whereas 1T’-MoS${}_{2}$ phase is a disturbed 1T-MoS${}_{2}$ phase [6].

The most accurate methods of solid state physics are based on quantum mechanics, unfortunately, with the accuracy of the methods their cost increases. The number of atoms and the number of timesteps that can be analysed with the first-principles method using either energy minimisation or ab initio molecular dynamics (AIMD) is highly limited. For typical computational resources currently available, the use of these methods is limited to several hundreds of atoms for less than about several picoseconds. These restrictions justify the need for more approximate methods, such as molecular methods [7].

In general, there is a lack of perfectly transferable interatomic potential that would work with the various materials and systems we are interested in. Some are more transferable, others less [8]. It depends on the physics behind them, the mathematical flexibility of the model capable of describing the multimodal potential energy surface (PES) and the quality of the fitting process and, of course, on the “difficulty” of the material [7].

According to the author’s best knowledge, there are no publications where the performance of different molecular potentials for molybdenum disulphide is analysed for all phases of SL MoS${}_{2}$, there are only partial comparisons, and so in [9] the results for 1H and 1T phases for potentials Stillinger–Weber, REBO and ReaxFF are only compared between each other. In [10], the geometric parameters and mechanical properties of 1H phase obtained from Stillinger–Weber and REBO potentials are compared with density functional theory (DFT) calculations. Thermal transport properties in 1H phase from molecular dynamics using Stillinger–Weber and REBO potentials were obtained in [11].

A partial comparison of different potentials for the 1H phase SL MoS${}_{2}$ can be found in papers where new parametrisations are presented, e.g., [12,13,14]. There are also publications where using molecular simulations the authors try to determine certain SL MoS${}_{2}$ properties that were not taken into account during the parametrization of potential, e.g., [15,16,17,18].

The paper is organised as follows. Following the above Section 1, Section 2.1 presents the computational methodology used in ab initio calculations of analysed structures and Section 2.2 describes the computational methodology used in molecular calculations and molecular potentials examined: four Stillinger–Weber (SW) potentials [19], the reactive many-body (REBO) potential, the spectral neighbour analysis potential (SNAP) and the reactive force-field (ReaxFF). Section 3 presents the structural and mechanical properties of SL MoS${}_{2}$ and phonon spectra obtained from the ab initio and molecular calculations and evaluates the quality of the analysed potentials. The last Section 4 summarises and concludes the results obtained.

## 2. Computational Methodology

Analysing the available literature concerning phases of SL MoS${}_{2}$, it is not feasible to find all structural, mechanical and phonon data obtained in one consistent way. The availability of experimental data is actually limited to phase 1H only and therefore we must use ab initio calculations. Unfortunately, also ab initio calculations, most often DFT, differ in the calculation methodology, i.e., they use different functional bases, different pseudopotential or exchange-correlation (XC) functionals, and such a parameter as cohesive energy is not accessible at all. For this reason, structural and mechanical data—lattice parameters, average cohesive energy, average bond length, average height, 2D elastic constants as well as phonon data—are determined using a single consistent first-principle approach as described in the next Section 2.1. These data will be further considered as reference data and marked as ${\mathrm{Value}}^{\mathrm{DFT}}$. Then the same data were determined, as described in Section 2.2, using the analysed molecular potentials Section 2.2 and will be marked as ${\mathrm{Value}}^{\mathrm{potential}}$. Having both data, we can simply define mean absolute percentage error (MAPE):

(1) $$\mathrm{MAPE}=\frac{100\%}{n}\sum_{t=1}^{n}\left|\frac{{\mathrm{Value}}^{\mathrm{DFT}}-{\mathrm{Value}}^{\mathrm{potential}}}{{\mathrm{Value}}^{\mathrm{DFT}}}\right|,$$

that will allow us to quantify the potentials under examination. Phonons were determined only for the three best, having the lowest ∑MAPE, molecular potentials.

### 2.1. Ab Initio Calculations

Ab initio computations by means of the density functional theory (DFT) [20,21] and the pseudopotential plane-wave approximation (PP-PW) programmed in ABINIT [22,23] code were done in the present study. Optimised norm-conserving Vanderbilt pseudopotentials (ONCVPSP) [24] were utilised to describe the interactions of non-valence electrons and ionic core. ONCVPSP pseudopotentials used were taken from PseudoDojo project [25].

To strengthen the reliability of the calculations as an exchange-correlation (XC) functional, three approximations were initially checked for their ability to reproduce the geometry of 1H-MoS${}_{2}$: local density approximation (LDA) [26,27], classical Perdew–Burke–Ernzerhof (PBE) generalised gradient approximation (GGA) [28] and modified Perdew–Burke–Ernzerhof GGA for solids (PBEsol) [29]. To provide access to all XC functionals used a library of exchange-correlation functionals for density functional theory, LibXC [30] was utilised.

All the computations were done by a proper adjustment of their precision, what was achieved by automatically set up the variables at *accuracy* level 4 (*accuracy* = 4 matches the default settings of ABINIT). The *cut-off* energy in line with ONCVPSP pseudopotentials of the plane-wave basis set was fixed at 35 Ha (952.4 eV) with $4d^{5}5s^{1}$ valence electrons for Mo and $3s^{2}3p^{4}$ valence electrons for S. K-PoinTs grids were derived with *kptrlen* = 35.0 (grids that specify a length of the smallest vector LARGER than *kptrlen*). In all the present ABINIT computations, the metallic occupation of levels with the Fermi–Dirac smearing occupation scheme and *tsmear* (Ha) = 0.02 was applied.

Initial data defining unit cells of SL 1H-MoS${}_{2}$, 1T-MoS${}_{2}$ and 1T’-MoS${}_{2}$ were taken from [31] and then all structures were relaxed by applying the BFGS minimisation scheme with full optimisation of cell geometry and atomic coordinates (a two-stage scheme was used here: in the first one, the ionic positions without cell shape and size optimization, and in the second, the full optimization of cell geometry). Tolerance for maximum stress (GPa) was specified as 1 × 10${}^{-4}$.

The cohesive energy the $E_{c}\left(MoS_{2}\right)$ was calculated, taking into account stoichiometry, as the total energy $E_{total}\left(MoS_{2}\right)$ difference of 2D molybdenum disulphide and a single Mo atom energy $E_{iso}\left(Mo\right)$ in a sufficiently large box and a single S atom energy $E_{iso}\left(S\right)$ in a similar large box [32]:

(2) $$\begin{array}{c} E_{c}\left({MoS}_{2}\right)=E_{total}\left({MoS}_{2}\right)-E_{iso}\left(Mo\right)-2E_{iso}\left(S\right). \end{array}$$

The theoretical ground state elasticity tensor, $C_{ij}$, of all the structures analysed, was identified with the metric tensor formulation of strain in density functional perturbation theory (DFPT) [33].

In order to examine the elastic (mechanical, Born) stability of all the structures, positive definiteness of the elasticity tensor was verified [34] by computing Kelvin moduli, i.e., eigenvalues of the elasticity tensor represented in second-rank tensor notation [35,36].

To compute phonons, DFPT was used [22,23]. The phonon dispersion curves (for 1H-MoS${}_{2}$ and 1T-MoS${}_{2}$: Γ[0,0,0]- **M**[1/2,0,0]-**K**[1/3,1/3,0]-Γ[0,0,0], and for 1T’-MoS${}_{2}$: Γ[0,0,0]-**Z**[0,1/2,0]-**C**[1/2,1/2,0]-**Y**[1/2,0,0]-Γ[0,0,0]) [37] of the structures examined were then utilised to identify their dynamical stability [34,38], complementary to the elastic stability.

### 2.2. Molecular Calculations

The molecular statics (MS) method (i.e., at 0 K temperature) [7,39,40] simulations were made using the Large-scale Atomic/Molecular Massively Parallel Simulator (LAMMPS) [41] and analysed in the Open Visualization Tool (OVITO) [42].

To get the elastic constants, $C_{ij}$, for all pre-relaxed structures, the stress–strain method with the maximum strain amplitude of 10${}^{-6}$ was utilised [41,43].

For the phonon calculations, phonoLAMMPS (LAMMPS interface for phonon calculations using Phonopy code [44]) [45] was utilised. Supercell and finite displacement approaches were used with 3 × 3 × 1 supercell of the unit cell and the atomic displacement distance of 0.01 Å. The cohesive energy, $E_{c}\left(MoS_{2}\right)$, (Equation (2)) in molecular calculations is simply potential energy.

#### Molecular Potentials

- **SW2013** [13]: the Stillinger–Weber (SW) potential fitted to an experimentally obtained phonon spectrum along the Γ-M direction for bulk 2H-MoS${}_{2}$.
- **SW2015** [14]: the Stillinger–Weber (SW) potential derived from the valence force-field model.
- **SW2016** [46]: the Stillinger–Weber (SW) potential fitted to lattice parameters, distance between two chalcogen atoms and elastic constants for SL 1H-MoS${}_{2}$ obtained from DFT calculations.
- **SW2017** [12,47]: the force-matching Stillinger–Weber (SW) potential fitted to first principles forces for a training set of atomic configurations of SL 1H-MoS${}_{2}$.
- **REBO** [48]: the reactive many-body potential (REBO) fitted to structure and energetics of Mo molecules, three-dimensional Mo crystals, two-dimensional Mo structures, small S molecules and binary Mo-S crystal structures.
- **SNAP** [49]: the machine-learning-based spectral neighbour analysis potential (SNAP) fitted to total energies and interatomic forces in SL 1H-MoS${}_{2}$ obtained from first-principles density functional theory (DFT) calculations.
- **ReaxFF** [50]: the reactive force-field (ReaxFF) parameters fitted to a training set of geometries, energies, and charges derived from DFT calculations for both clusters and periodic Mo${}_{x}$S${}_{y}$ systems.

## 3. Results

The first step of the ab initio calculation was to select the exchange-correlation (XC) functional that most accurately reproduces the experimental geometry of 1H-MoS${}_{2}$. The measured lattice constant for SL 1H-MoS${}_{2}$ a = 3.157 Å and average height (vertical separation between S atoms) *h* = 3.116 Å [50], while that calculated with the local density approximation (LDA) a = 3.144 Å, *h* = 3.111 Å, with the classical Perdew–Burke–Ernzerhof (PBE) generalised gradient approximation (GGA) a = 3.220 Å, *h* = 3.121 Å and with the modified Perdew–Burke–Ernzerhof GGA for solids (PBEsol) a = 3.165 Å, *h* = 3.120 Å, a similar trend can also be observed in other papers [12,51]. Once again, it was confirmed that the PBEsol is the overall best performing XC functional for identifying the structural and mechanical properties [4,52,53] and thus all subsequent calculations will use PBEsol XC functional.

### 3.1. Structural and Mechanical Properties

**Figure 1:**
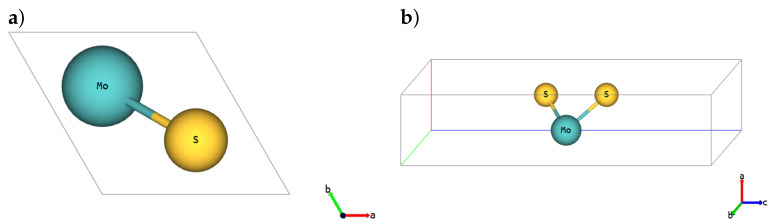
Single-layer (SL) 1H-MoS${}_{2}$. (**a**) Top and (**b**) 3D view.

**Figure 2:**
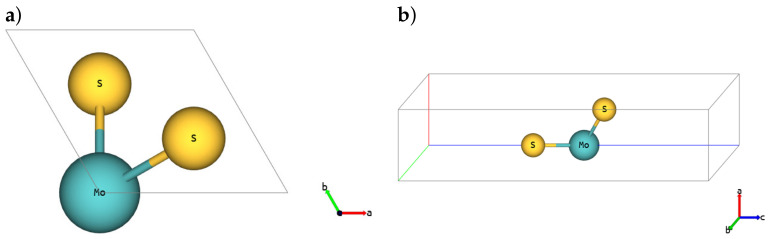
SL 1T-MoS${}_{2}$. (**a**) Top and (**b**) 3D view.

**Figure 3:**
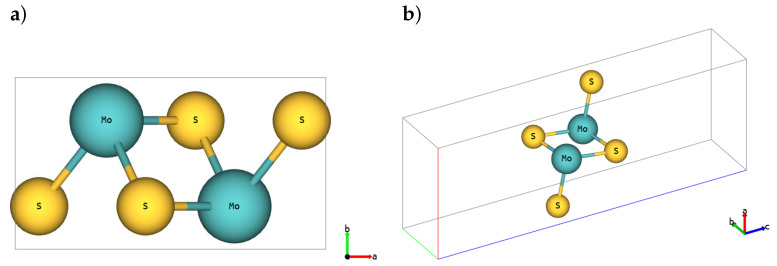
SL 1T’-MoS${}_{2}$. (**a**) Top and (**b**) 3D view.

The basic cell for the SL 1H-MoS${}_{2}$ polymorph is depicted in Figure 1 (*hP3* in Pearson notation, P$\bar{6}$m2-space group in Hermann–Mauguin notation, no.187-space group in the International Union of Crystallography (IUCr) notation), SL 1T-MoS${}_{2}$ polymorph is shown in Figure 2 (*hP3*, P$\bar{3}$m1, no.164) and SL 1T’-MoS${}_{2}$ is depicted in Figure 3 (*oP6*, P2${}_{1}$/m, no.11), respectively [54]. Although 2D structures were studied, as is commonly practised, the 3D notation is used here. The crystallographic data for all calculated phases are additionally stored in crystallographic information files (CIFs) in Appendix A.

Determined from DFT calculations structural and mechanical properties, namely, lattice parameters, average cohesive energy, average bond length, average height, 2D elastic constants and 2D Kelvin moduli, of the three analysed SL MoS${}_{2}$ allotropes are gathered in Table 1. It can be seen that the calculated values match well the available experimental data [50] as well as those from other calculations [55,56,57]. This can be regarded as a confirmation of the correctness of the applied methodology. It is worth observing that the trend in the calculated cohesive energy matches the stability of the analysed phases and adding that all the calculated values are obtained using one consistent methodological approach. All calculated 2D Kelvin moduli for the three analysed phases are positive, which translates into mechanical stability.

Calculated with the use of molecular statics and different molecular potentials, twelve structural and mechanical properties, namely, lattice parameters, average cohesive energy, average bond length, average height, 2D elastic constants and 2D Kelvin moduli, of the SL 1H-MoS${}_{2}$ phase are collected in Table 2. The results obtained are then compared with those from DFT and quantified by calculating the mean absolute percentage error (MAPE) using the Equation (1). What follows from the results obtained? Overall, analysing the MAPE${}_{1H}$ for 1H-MoS${}_{2}$, the three most accurate potentials are: SW2017, SNAP, REBO, and the least are: ReaxFF and SW2015. A detailed look shows that only two potentials correctly reproduce cohesive energy. Mechanical stability is correctly reproduced by all potentials, i.e., $K_{i}>0$. Potential ReaxFF catastrophically badly reproduces mechanical properties of 1H-MoS${}_{2}$, even the symmetry of the elasticity tensor is not correct.

The computed twelve structural and mechanical properties of the SL 1T-MoS${}_{2}$ phase are summarised in Table 3. In general, analysing the MAPE${}_{1T}$ for 1T-MoS${}_{2}$, the three most accurate potentials are: SW2015, SW2016 and SW2017, and the least are: SNAP and ReaxFF. A detailed look shows that only two potentials correctly reproduce cohesive energy. Mechanical stability is correctly reproduced by all potentials, i.e., $K_{i}>0$. Potential ReaxFF again catastrophically badly reproduces mechanical properties of 1T-MoS${}_{2}$, even the symmetry of the stiffness tensor is again not correct. Unfortunately, the three potentials: SW2013, SW2015, SW2016, do not correctly reproduce the symmetry of the 1T-MoS${}_{2}$ phase, i.e., during pre-relaxation input 1T-MoS${}_{2}$ converges to 1H-MoS${}_{2}$ phase.

The identified thirteen structural and mechanical properties of the SL 1T’-MoS${}_{2}$ phase are summarised in Table 4. In general, analysing the MAPE${}_{1T^{\prime }}$ for 1T’-MoS${}_{2}$, the three most accurate potentials are: SW2016, REBO and SW2017, and the least are: SNAP and ReaxFF. Once again, only two potentials correctly reproduce cohesive energy. Mechanical stability is reproduced in the right way by all potentials, i.e., $K_{i}>0$. Unfortunately, the two potentials, SW2017 and SNAP, do not properly restore the symmetry of the 1T’-MoS${}_{2}$ phase, i.e., during pre-relaxation input 1T’-MoS${}_{2}$ basic cell converges to 1T-MoS${}_{2}$.

Let us now analyse the cumulative performance of the analysed potentials for all SL MoS${}_{2}$ phases. We see that ∑MAPE in Table 4 is the lowest, and almost the same, for three potentials: SW2017, SW2016 and REBO. However, only the REBO potential distinguishes three different phases, the other two potentials degenerate phases, i.e., instead of three they produce two.

### 3.2. Phonon Spectra

**Figure 4:**
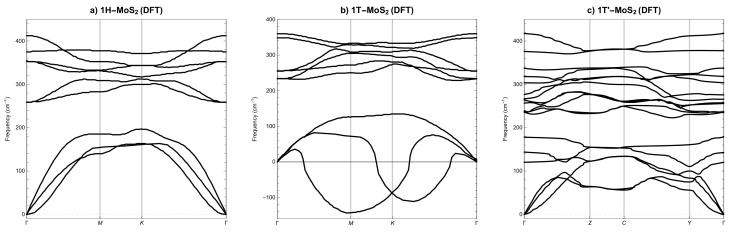
Phonon dispersion of SL MoS${}_{2}$ from DFT (**a**) 1H, (**b**) 1T and (**c**) 1T’. High symmetry points: Γ[0,0,0], **M**[1/2,0,0], **K**[1/3,1/3,0], **Z**[0,1/2,0], **C**[1/2,1/2,0], **Y**[1/2,0,0].

Phonon spectra along the appropriate high symmetry q-points [37], calculated by applying the PBEsol XC functional, for SL 1H-MoS${}_{2}$ phase are depicted in Figure 4a), for SL 1T-MoS${}_{2}$ phase are depicted in Figure 4b) and for SL 1T’-MoS${}_{2}$ phase are shown in Figure 4c), respectively. Experimental data for single-layer molybdenum disulphide are very scarce and concern only Γ point in 1H-MoS${}_{2}$ phase, see [58]. When we compare the results obtained here with those calculated by other authors, we see agreement typical for different DFT calculations, see [31,59,60,61,62].

Analysis of the computed curves in Figure 4a–c allows us to conclude that phases 1H-MoS${}_{2}$ and 1T’-MoS${}_{2}$ are not only mechanically but also dynamically stable, i.e., phonon modes everywhere have positive frequencies. Phase 1T-MoS${}_{2}$ is mechanically stable, but not dynamically stable, i.e., phonon modes also have negative frequencies. Similar observations can be found in [31,60].

**Figure 5:**
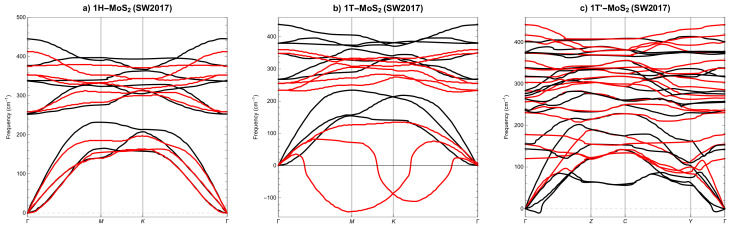
Phonon dispersion of SL MoS${}_{2}$ from SW2017 potential (**a**) 1H, (**b**) 1T and (**c**) 1T’. Black lines represent SW2017 results, red lines represent DFT results.

**Figure 6:**
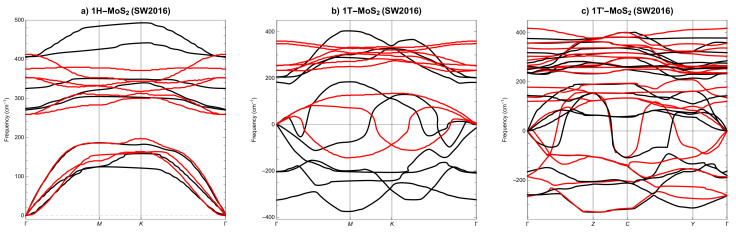
Phonon dispersion of SL MoS${}_{2}$ from SW2016 potential (**a**) 1H, (**b**) 1T and (**c**) 1T′. Black lines represent SW2016 results, red lines represent DFT results.

**Figure 7:**
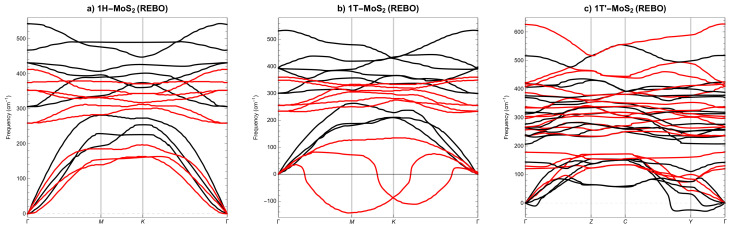
Phonon dispersion of SL MoS${}_{2}$ from reactive many-body (REBO) potential (**a**) 1H, (**b**) 1T and (**c**) 1T′. Black lines represent REBO results, red lines represent DFT results.

Let us now compare the phonon spectra for SL MoS${}_{2}$ phases calculated with DFT, Figure 4, and those calculated with LAMMPS and the three best potentials, i.e., SW2017, Figure 5, SW2016, Figure 6 and REBO, Figure 7. As a result that only the REBO potential distinguishes three different phases of SL MoS${}_{2}$, the molecular calculations of phonons utilise basic cells derived from DFT calculations. At first glance, we can see that it only makes sense to compare molecular phonons with DFT phonons merely qualitatively, not quantitatively. All three potentials are qualitatively well reproducing the phonon spectra for SL 1H-MoS${}_{2}$ phase, see Figure 5a, Figure 6a and Figure 7a. For SL 1T-MoS${}_{2}$ phase, only SW2016 potential predicts dynamical instability, see Figure 6b. For SL 1T’-MoS${}_{2}$ phase, SW2017 and REBO potentials behave reasonably, see Figure 5c and Figure 7c. The conclusion is that none of the three potentials correctly reproduces the dynamical stability of all SL MoS${}_{2}$ phases.

## 4. Conclusions

A systematic quantitative comparison of Stillinger–Weber, REBO, SNAP and ReaxFF potentials for the reproduction of the properties of 2D molybdenum disulphide polymorphs was presented. To compare the potentials, the structural and mechanical properties and phonon dispersion of single-layer phases 1H, 1T and 1T’ MoS${}_{2}$ (SL MoS${}_{2}$) obtained from the functional density theory (DFT) and molecular static (MS) calculations were used.

We can conclude that:

- The transferability of analysed molecular potentials leaves much to be desired.
- Three potentials: SW2016, SW2017 and REBO demonstrate the best quantitative performance.
- None of the above three potentials correctly reproduces the dynamical stability of all SL MoS${}_{2}$ phases.
- Only the REBO potential distinguishes three different 2D molybdenum disulphide allotropes.
- Two potentials, ReaxFF and SNAP, demonstrate significantly lower quantitative efficiency.
- It seems that the low transferability of the analysed potentials is a result of the improper fitting of their parameters.
- To increase the transferability of potentials, the number of configurations to be taken into account in the parameter optimisation process should be significantly increased.

I hope that the observations made here will help other researchers to choose the right potentials for their purposes and will be a suggestion for parametrising new potentials for SL MoS${}_{2}$.

## Figures and Tables

**Table 1:** Structural and mechanical properties of SL MoS${}_{2}$ phases from density functional theory (DFT) calculations: lattice parameters a,b (Å), average cohesive energy $E_{c}$ (eV/atom), average bond length *d* (Å), average height *h* (Å), 2D elastic constants $C_{ij}$ (N/m) and 2D Kelvin moduli $K_{i}$ (N/m).

Polymorph	1H			1T			1T′		
**Source**	**Present**	**Exp.**	**DFT**	**Present**	**Exp.**	**DFT**	**Present**	**Exp.**	**DFT**
a	3.165	3.157 ^a^	3.183 ^b^	3.194		3.179 ^b^	5.751		5.717 ^b^
b	3.165	3.157 ^a^	3.183 ^b^	3.194		3.176 ^b^	3.177		3.179 ^b^
$-E_{c}$	5.64		5.35 ^a^	5.52			5.56		
$d_{Mo-S}$	2.403	2.38 ^a^	2.43 ^a^	2.422		2.430 ^c^	2.415 ^‡^		
$h_{S-S}$	3.120	3.116 ^a^	3.11 ^a^	3.142		3.184 ^c^	3.364		
$C_{11}$	126.5		127.2 ^d^	84.1		103.8 ^d^	68.1		94.0 ^d^
$C_{22}$	126.5		127.2 ^d^	84.1		103.8 ^d^	78.9		119.2 ^d^
$C_{12}$	28.5		25.8 ^d^	5.0		−2.5 ^d^	18.2		17.2 ^d^
$C_{44}$	49.0		51.0 ^d^	39.6		52.8 ^d^	43.2		37.5 ^d^
$K_{I}$	155.0			89.1			90.9		
$K_{II}$	98.0			79.1			56.1		
$K_{III}$	98.0			79.1			86.4		

^a^ Ref. [50], ^b^ Ref. [55], ^c^ Ref. [56], ^d^ Ref. [57], ^‡^ average first-neighbour bond lengths calculated with *cutoff* radius = 3.5 and number of histogram bins = 50.

**Table 2:** Structural and mechanical properties of SL 1H-MoS${}_{2}$ from molecular calculations: lattice parameters a,b (Å), average cohesive energy $E_{c}$ (eV/atom), average bond length *d* (Å), average height *h* (Å), 2D elastic constants $C_{ij}$ (N/m), 2D Kelvin moduli $K_{i}$ (N/m), mean absolute percentage error (MAPE) (%).

Method	DFT	SW2013	SW2015	SW2016	SW2017	REBO	SNAP	ReaxFF
a	3.165	3.062	3.117	3.174	3.196	3.168	3.139	3.186
b	3.165	3.062	3.117	3.174	3.196	3.168	3.139	3.186
$-E_{c}$	5.64	3.00	0.62	1.84	5.11	7.16	2.28	5.05
$d_{Mo-S}$	2.403	2.399	2.382	2.515	2.441	2.445	2.392	2.431
$h_{S-S}$	3.120	4.223	4.257	4.032	3.194	3.242	3.124	3.183
$C_{11}$	126.5	103.9	45.8	90.0	118.9	154.4	140.3	237.3
$C_{22}$	126.5	103.9	45.8	90.0	118.9	154.4	140.3	262.4
$C_{12}$	28.5	33.4	8.0	30.1	40.9	45.8	35.7	121.2
$C_{44}$	49.0	35.2	18.9	30.0	39.0	54.3	52.3	71.2
$K_{I}$	155.0	137.3	53.8	120.1	159.8	200.2	176.0	370.4
$K_{II}$	98.0	70.5	37.8	59.9	78.0	108.6	104.6	129.3
$K_{III}$	98.0	70.4	37.8	60.0	78.0	108.6	104.6	142.4
MAPE${}_{1H}$		19.797	48.204	25.342	11.263	16.602	11.886	66.398

**Table 3:** Structural and mechanical properties of SL 1T-MoS${}_{2}$ from molecular calculations: lattice parameters a,b (Å), average cohesive energy $E_{c}$ (eV/atom), average bond lengths *d* (Å), average height *h* (Å), 2D elastic constants $C_{ij}$ (N/m), 2D Kelvin moduli $K_{i}$ (N/m), mean absolute percentage error (MAPE) (%).

Method	DFT	SW2013	SW2015	SW2016	SW2017	REBO	SNAP	ReaxFF
a	3.194	3.062 *	3.117 *	3.174 *	3.307	3.194	3.072	3.162
b	3.194	3.062 *	3.117 *	3.174 *	3.307	3.194	3.072	3.162
$-E_{c}$	5.52	3.00	0.62	1.84	4.96	7.05	2.31	4.84
$d_{Mo-S}$	2.422	2.399	2.382	2.515	2.42	2.445	2.476	2.433
$h_{S-S}$	3.142	4.223	4.257	4.032	2.973	3.211	3.454	3.203
$C_{11}$	84.1	103.9	45.8	91.7	121.8	118.2	437.1	173.3
$C_{22}$	84.1	103.9	45.8	91.7	121.8	118.2	437.1	32.1
$C_{12}$	5.0	33.4	8.0	28.4	28.6	32.4	6.1	83.8
$C_{44}$	39.6	35.2	18.9	31.7	46.6	42.9	215.5	9.4
$K_{I}$	89.1	137.3	53.8	120.1	150.4	150.6	443.2	147.8
$K_{II}$	79.1	70.5	37.8	63.3	93.2	85.8	431.0	57.6
$K_{III}$	79.2	70.4	37.8	63.4	93.2	85.8	431.0	18.8
MAPE${}_{1T}$		65.962	39.849	56.735	58.860	62.843	222.509	167.192

^*^ Input 1T converges to 1H.

**Table 4:** Structural and mechanical properties of SL 1T′-MoS${}_{2}$ from molecular calculations: lattice parameters a,b (Å), average cohesive energy $E_{c}$ (eV/atom), average bond lengths *d* (Å), average height *h* (Å), 2D elastic constants $C_{ij}$ (N/m), 2D Kelvin moduli $K_{i}$ (N/m), mean absolute percentage error (MAPE) (%).

Method	DFT	SW2013	SW2015	SW2016	SW2017	REBO	SNAP	ReaxFF
a	5.751	4.944	5.757	5.263	5.728 ^†^	5.563	5.321 ^†^	5.609
b	3.177	3.062	3.148	3.172	3.307 ^†^	3.245	3.072 ^†^	3.209
$-E_{c}$	5.56	3.02	0.55	1.87	4.96	6.93	2.31	4.83
$d_{Mo-S}$ ^‡^	2.415	2.399	2.406	2.504	2.42	2.468	2.476	2.490
$h_{S-S}$	3.364	4.641	5.173	4.142	2.973	3.781	3.454	3.399
$C_{11}$	68.1	1.1	0.0	60.4	121.8	56.8	437.1	120.1
$C_{22}$	78.9	100.5	37.6	94.6	121.8	113.0	437.1	255.7
$C_{12}$	18.2	1.1	0.0	20.3	28.6	23.1	6.1	68.1
$C_{44}$	43.2	27.1	0.0	26.9	46.6	70.5	215.5	6.4
$K_{I}$	90.9	100.5	37.6	88.4	150.4	121.3	443.2	194.3
$K_{II}$	56.1	1.1	0.0	66.6	93.2	48.5	431.0	181.5
$K_{III}$	86.4	54.2	0.0	53.8	93.2	141.0	431.0	12.8
MAPE${}_{1T^{\prime }}$		42.070	63.020	20.110	30.399	25.395	249.177	91.913
∑MAPE		127.830	151.074	102.187	100.522	104.840	483.573	325.504

^†^ Input 1T′ converges to 1H. ^‡^ average first-neighbour bond lengths calculated with *cutoff* radius = 3.5 and number of histogram bins = 50.

## Abbreviations

The following abbreviations are used in this manuscript:

G6-TMD	Group 6 transition metal dichalcogenide
SL MoS${}_{2}$	single-layer molybdenum disulphide
MS	molecular statics
DFT	density functional theory
DFPT	density functional perturbation theory
PP-PW	pseudopotential, plane-wave
XC	exchange-correlation
LDA	local density approximation
GGA	generalized gradient approximation
PBE	Perdew–Burke–Ernzerhof

## Appendix A

# CIF file 1H-MoS2

# This file was generated by FINDSYM

# Harold T. Stokes, Branton J. Campbell, Dorian M. Hatch

# Brigham Young University, Provo, Utah, USA

data_findsym-output

_audit_creation_method FINDSYM

_symmetry_space_group_name_H-M "P -6 m 2"

_symmetry_Int_Tables_number 187

_cell_length_a    3.16544

_cell_length_b    3.16544

_cell_length_c    16.00000

_cell_angle_alpha 90.00000

_cell_angle_beta  90.00000

_cell_angle_gamma 120.00000

loop_

_space_group_symop_id

_space_group_symop_operation_xyz

1 x,y,z

2 -y,x-y,z

3 -x+y,-x,z

4 x,x-y,-z

5 -x+y,y,-z

6 -y,-x,-z

7 -x+y,-x,-z

8 x,y,-z

9 -y,x-y,-z

10 -x+y,y,z

11 -y,-x,z

12 x,x-y,z

loop_

_atom_site_label

_atom_site_type_symbol

_atom_site_symmetry_multiplicity

_atom_site_Wyckoff_label

_atom_site_fract_x

_atom_site_fract_y

_atom_site_fract_z

_atom_site_occupancy

Mo1 Mo  1 d 0.33333 0.66667 0.50000 1.00000

S1 S  2 i 0.66667 0.33333 0.40249 1.00000

# CIF file 1T-MoS2

# This file was generated by FINDSYM

# Harold T. Stokes, Branton J. Campbell, Dorian M. Hatch

# Brigham Young University, Provo, Utah, USA

data_findsym-output

_audit_creation_method FINDSYM

_symmetry_space_group_name_H-M "P -3 2/m 1"

_symmetry_Int_Tables_number 164

_cell_length_a    3.19358

_cell_length_b    3.19358

_cell_length_c    16.00000

_cell_angle_alpha 90.00000

_cell_angle_beta  90.00000

_cell_angle_gamma 120.00000

loop_

_space_group_symop_id

_space_group_symop_operation_xyz

1 x,y,z

2 -y,x-y,z

3 -x+y,-x,z

4 x-y,-y,-z

5 y,x,-z

6 -x,-x+y,-z

7 -x,-y,-z

8 y,-x+y,-z

9 x-y,x,-z

10 -x+y,y,z

11 -y,-x,z

12 x,x-y,z

loop_

_atom_site_label

_atom_site_type_symbol

_atom_site_symmetry_multiplicity

_atom_site_Wyckoff_label

_atom_site_fract_x

_atom_site_fract_y

_atom_site_fract_z

_atom_site_occupancy

Mo1 Mo  1 b 0.00000 0.00000 0.50000 1.00000

S1 S  2 d 0.33333 0.66667 0.40182 1.00000

# CIF file 1T’-MoS2

# This file was generated by FINDSYM

# Harold T. Stokes, Branton J. Campbell, Dorian M. Hatch

# Brigham Young University, Provo, Utah, USA

data_findsym-output

_audit_creation_method FINDSYM

_symmetry_space_group_name_H-M "P 1 21/m 1"

_symmetry_Int_Tables_number 11

_cell_length_a    5.75123

_cell_length_b    3.17711

_cell_length_c    16.00000

_cell_angle_alpha 90.00000

_cell_angle_beta  90.00000

_cell_angle_gamma 90.00000

loop_

_space_group_symop_id

_space_group_symop_operation_xyz

1 x,y,z

2 -x,y+1/2,-z

3 -x,-y,-z

4 x,-y+1/2,z

loop_

_atom_site_label

_atom_site_type_symbol

_atom_site_symmetry_multiplicity

_atom_site_Wyckoff_label

_atom_site_fract_x

_atom_site_fract_y

_atom_site_fract_z

_atom_site_occupancy

Mo1 Mo  2 e 0.70568 0.25000 0.49753 1.00000

S1 S  2 e 0.41977 0.25000 0.60514 1.00000

S2 S  2 e 0.07725 0.25000 0.41677 1.00000

## References

[1] Samadi M., Sarikhani N., Zirak M., Zhang H., Zhang H.L., Moshfegh A.Z. (2018). Group 6 transition metal dichalcogenide nanomaterials: Synthesis, applications and future perspectives. Nanoscale Horiz..

[2] Geim A., Novoselov K. (2007). The rise of graphene. Nat. Mater..

[3] Manzeli S., Ovchinnikov D., Pasquier D., Yazyev O.V., Kis A. (2017). 2D transition metal dichalcogenides. Nat. Rev. Mater..

[4] Maździarz M., Mrozek A., Kuś W., Burczyński T. (2018). *Anisotropic-Cyclicgraphene*: A New Two-Dimensional Semiconducting Carbon Allotrope. Materials.

[5] Zhao X., Ning S., Fu W., Pennycook S.J., Loh K.P. (2018). Differentiating Polymorphs in Molybdenum Disulfide via Electron Microscopy. Adv. Mater..

[6] Esteban-Puyuelo R., Sarma D.D., Sanyal B. (2020). Complexity of mixed allotropes of *MoS*_2_ unraveled by first-principles theory. Phys. Rev. B.

[7] Tadmor E.B., Miller R.E. (2011). Modeling Materials: Continuum, Atomistic and Multiscale Techniques.

[8] Rowe P., Deringer V.L., Gasparotto P., Csányi G., Michaelides A. (2020). An accurate and transferable machine learning potential for carbon. J. Chem. Phys..

[9] Mrozek A. (2019). Basic mechanical properties of 2h and 1t single-layer molybdenum disulfide polymorphs. a short comparison of various atomic potentials. Int. J. Multiscale Comput. Eng..

[10] Xiong S., Cao G. (2015). Molecular dynamics simulations of mechanical properties of monolayer MoS_2_. Nanotechnology.

[11] Xu K., Gabourie A.J., Hashemi A., Fan Z., Wei N., Farimani A.B., Komsa H.P., Krasheninnikov A.V., Pop E., Ala-Nissila T. (2019). Thermal transport in *MoS*_2_ from molecular dynamics using different empirical potentials. Phys. Rev. B.

[12] Wen M., Shirodkar S.N., Plecháč P., Kaxiras E., Elliott R.S., Tadmor E.B. (2017). A force-matching Stillinger-Weber potential for MoS_2_: Parameterization and Fisher information theory based sensitivity analysis. J. Appl. Phys..

[13] Jiang J.W., Park H.S., Rabczuk T. (2013). Molecular dynamics simulations of single-layer molybdenum disulphide (MoS_2_): Stillinger-Weber parametrization, mechanical properties, and thermal conductivity. J. Appl. Phys..

[14] Jiang J.W. (2015). Parametrization of Stillinger–Weber potential based on valence force field model: Application to single-layer MoS_2_ and black phosphorus. Nanotechnology.

[15] Mortazavi B., Ostadhossein A., Rabczuk T., van Duin A. (2016). Mechanical response of all-MoS_2_ single-layer hetrostructures: A ReaxFF investigation. Phys. Chem. Chem. Phys..

[16] Bao H., Huang Y., Yang Z., Sun Y., Bai Y., Miao Y., Chu P.K., Xu K., Ma F. (2018). Molecular Dynamics Simulation of Nanocrack Propagation in Single-Layer MoS_2_ Nanosheets. J. Phys. Chem. C.

[17] Pang H., Li M., Gao C., Huang H., Zhuo W., Hu J., Wan Y., Luo J., Wang W. (2018). Phase Transition of Single-Layer Molybdenum Disulfide Nanosheets under Mechanical Loading Based on Molecular Dynamics Simulations. Materials.

[18] Javeed Akhter M., Kuś W., Mrozek A., Burczyński T. (2020). Mechanical Properties of Monolayer MoS_2_ with Randomly Distributed Defects. Materials.

[19] Stillinger F.H., Weber T.A. (1985). Computer simulation of local order in condensed phases of silicon. Phys. Rev. B.

[20] Hohenberg P., Kohn W. (1964). Inhomogeneous electron gas. Phys. Rev..

[21] Kohn W., Sham L.J. (1965). Self-consistent equations including exchange and correlation effects. Phys. Rev..

[22] Gonze X., Jollet F., Araujo F.A., Adams D., Amadon B., Applencourt T., Audouze C., Beuken J.M., Bieder J., Bokhanchuk A. (2016). Recent developments in the ABINIT software package. Comput. Phys. Commun..

[23] Gonze X., Amadon B., Antonius G., Arnardi F., Baguet L., Beuken J.M., Bieder J., Bottin F., Bouchet J., Bousquet E. (2020). The ABINIT project: Impact, environment and recent developments. Comput. Phys. Commun..

[24] Hamann D.R. (2013). Optimized norm-conserving Vanderbilt pseudopotentials. Phys. Rev. B.

[25] van Setten M., Giantomassi M., Bousquet E., Verstraete M., Hamann D., Gonze X., Rignanese G.M. (2018). The PseudoDojo: Training and grading a 85 element optimized norm-conserving pseudopotential table. Comput. Phys. Commun..

[26] Bloch F. (1929). Bemerkung zur Elektronentheorie des Ferromagnetismus und der elektrischen Leitfähigkeit. Zeitschrift für Physik.

[27] Perdew J.P., Wang Y. (1992). Accurate and simple analytic representation of the electron-gas correlation energy. Phys. Rev. B.

[28] Perdew J.P., Burke K., Ernzerhof M. (1996). Generalized Gradient Approximation Made Simple. Phys. Rev. Lett..

[29] Perdew J.P., Ruzsinszky A., Csonka G.I., Vydrov O.A., Scuseria G.E., Constantin L.A., Zhou X., Burke K. (2008). Restoring the Density-Gradient Expansion for Exchange in Solids and Surfaces. Phys. Rev. Lett..

[30] Lehtola S., Steigemann C., Oliveira M.J., Marques M.A. (2018). Recent developments in LIBXC—A comprehensive library of functionals for density functional theory. SoftwareX.

[31] Calandra M. (2013). Chemically exfoliated single-layer MoS_2_: Stability, lattice dynamics, and catalytic adsorption from first principles. Phys. Rev. B.

[32] Maździarz M., Mościcki T. (2016). Structural, mechanical, optical, thermodynamical and phonon properties of stable ReB_2_ polymorphs from density functional calculations. J. Alloys Compd..

[33] Hamann D.R., Wu X., Rabe K.M., Vanderbilt D. (2005). Metric tensor formulation of strain in density-functional perturbation theory. Phys. Rev. B.

[34] Grimvall G., Magyari-Köpe B., Ozoliņš V., Persson K.A. (2012). Lattice instabilities in metallic elements. Rev. Mod. Phys..

[35] Maździarz M. (2019). Comment on ‘The Computational 2D Materials Database: High-throughput modeling and discovery of atomically thin crystals’. 2D Mater..

[36] Maździarz M., Mościcki T. (2020). New Zirconium Diboride Polymorphs–First-Principles Calculations. Materials.

[37] Hinuma Y., Pizzi G., Kumagai Y., Oba F., Tanaka I. (2017). Band structure diagram paths based on crystallography. Comput. Mater. Sci..

[38] Řehák P., Černý M., Pokluda J. (2012). Dynamic stability of fcc crystals under isotropic loading from first principles. J. Phys. Condens. Matter.

[39] Maździarz M., Young T.D., Dłuzewski P., Wejrzanowski T., Kurzydłowski K.J. (2010). Computer modelling of nanoindentation in the limits of a coupled molecular–statics and elastic scheme. J. Comput. Theor. Nanosci..

[40] Maździarz M., Young T.D., Jurczak G. (2011). A study of the affect of prerelaxation on the nanoindentation process of crystalline copper. Arch. Mech..

[41] Plimpton S. (1995). Fast Parallel Algorithms for Short-Range Molecular Dynamics. J. Comput. Phys..

[42] Stukowski A. (2010). Visualization and analysis of atomistic simulation data with OVITO-the Open Visualization Tool. Model. Simul. Mater. Sci. Eng..

[43] Maździarz M., Gajewski M. (2015). Estimation of Isotropic Hyperelasticity Constitutive Models to Approximate the Atomistic Simulation Data for Aluminium and Tungsten Monocrystals. Comput. Model. Eng. Sci..

[44] Togo A., Tanaka I. (2015). First principles phonon calculations in materials science. Scr. Mater..

[45] Carreras A., Togo A., Tanaka I. (2017). DynaPhoPy: A code for extracting phonon quasiparticles from molecular dynamics simulations. Comput. Phys. Commun..

[46] Kandemir A., Yapicioglu H., Kinaci A., Çağın T., Sevik C. (2016). Thermal transport properties of MoS_2_ and MoSe_2_ monolayers. Nanotechnology.

[47] Tadmor E.B., Elliott R.S., Sethna J.P., Miller R.E., Becker C.A. (2011). The potential of atomistic simulations and the Knowledgebase of Interatomic Models. JOM.

[48] Liang T., Phillpot S.R., Sinnott S.B. (2012). Parametrization of a reactive many-body potential for Mo–S systems. Phys. Rev. B.

[49] Gu X., Zhao C. (2019). Thermal conductivity of single-layer MoS_2(1−*x*)_Se_2*x*_ alloys from molecular dynamics simulations with a machine-learning-based interatomic potential. Comput. Mater. Sci..

[50] Ostadhossein A., Rahnamoun A., Wang Y., Zhao P., Zhang S., Crespi V.H., van Duin A.C.T. (2017). ReaxFF Reactive Force-Field Study of Molybdenum Disulfide (MoS_2_). J. Phys. Chem. Lett..

[51] Antunes F.P.N., Vaiss V.S., Tavares S.R., Capaz R.B., Leitão A.A. (2018). Van der Waals interactions and the properties of graphite and 2H-, 3R- and 1T-MoS_2_: A comparative study. Comput. Mater. Sci..

[52] Råsander M., Moram M.A. (2015). On the accuracy of commonly used density functional approximations in determining the elastic constants of insulators and semiconductors. J. Chem. Phys..

[53] Maździarz M., Mrozek A., Kuś W., Burczyński T. (2017). First-principles study of new X-graphene and Y-graphene polymorphs generated by the two stage strategy. Mater. Chem. Phys..

[54] Stokes H.T., Hatch D.M. (2005). *FINDSYM*: Program for identifying the space-group symmetry of a crystal. J. Appl. Crystallogr..

[55] Duerloo K.A., Li Y., Reed E. (2014). Structural phase transitions in two-dimensional Mo- and W-dichalcogenide monolayers. Nat. Commun..

[56] Hu T., Li R., Dong J. (2013). A new (2 × 1) dimerized structure of monolayer 1T-molybdenum disulfide, studied from first principles calculations. J. Chem. Phys..

[57] Hung N.T., Nugraha A.R.T., Saito R. (2018). Two-dimensional MoS_2_ electromechanical actuators. J. Phys. D Appl. Phys..

[58] Luo X., Zhao Y., Zhang J., Xiong Q., Quek S.Y. (2013). Anomalous frequency trends in MoS_2_ thin films attributed to surface effects. Phys. Rev. B.

[59] Tornatzky H., Gillen R., Uchiyama H., Maultzsch J. (2019). Phonon dispersion in *MoS*_2_. Phys. Rev. B.

[60] Singh A., Shirodkar S.N., Waghmare U.V. (2015). 1H and 1T polymorphs, structural transitions and anomalous properties of (Mo,W)(S,Se)_2_ monolayers: First-principles analysis. 2D Mater..

[61] Soni H., Jha P.K. (2015). Ab-initio study of dynamical properties of two dimensional MoS_2_ under strain. AIP Adv..

[62] Molina-Sánchez A., Wirtz L. (2011). Phonons in single-layer and few-layer MoS_2_ and WS_2_. Phys. Rev. B.

